# Nanocarrier Drug Conjugates Exhibit Potent Anti-*Naegleria fowleri* and Anti-*Balamuthia mandrillaris* Properties

**DOI:** 10.3390/diseases11020058

**Published:** 2023-04-06

**Authors:** Ruqaiyyah Siddiqui, Anania Boghossian, Muhammad Kawish, Tooba Jabri, Muhammad Raza Shah, Tengku Shahrul Anuar, Zainab Al-Shareef, Naveed Ahmed Khan

**Affiliations:** 1 College of Arts and Sciences, American University of Sharjah, Sharjah 26666, United Arab Emirates; 2 Department of Medical Biology, Faculty of Medicine, Istinye University, Istanbul 34010, Turkey; 3 International Centre for Chemical and Biological Sciences, H.E.J. Research Institute of Chemistry, University of Karachi, Karachi 75270, Pakistan; 4 Centre for Medical Laboratory Technology Studies, Faculty of Health Sciences, Universiti Teknologi MARA, PuncakAlam Campus, Selangor 42300, Malaysia; 5 Department of Clinical Sciences, College of Medicine, University of Sharjah, Sharjah 27272, United Arab Emirates

**Keywords:** *Naegleria fowleri*, *Balamuthia mandrillaris*, curcumin, nanoparticle, anti-amoebic, fatal, blood brain barrier

## Abstract

Given the opportunity and access, pathogenic protists (*Balamuthia mandrillaris* and *Naegleria fowleri*) can produce fatal infections involving the central nervous system. In the absence of effective treatments, there is a need to either develop new antimicrobials or enhance the efficacy of existing compounds. Nanocarriers as drug delivery systems are gaining increasing attention in the treatment of parasitic infections. In this study, novel nanocarriers conjugated with amphotericin B and curcumin were evaluated for anti-amoebic efficacy against *B. mandrillaris* and *N. fowleri*. The results showed that nanocarrier conjugated amphotericin B exhibited enhanced cidal properties against both amoebae tested compared with the drug alone. Similarly, nanocarrier conjugated curcumin exhibited up to 75% cidal effects versus approx. 50% cidal effects for curcumin alone. Cytopathogenicity assays revealed that the pre-treatment of both parasites with nanoformulated-drugs reduced parasite-mediated host cellular death compared with the drugs alone. Importantly, the cytotoxic effects of amphotericin B on human cells alone were reduced when conjugated with nanocarriers. These are promising findings and further suggest the need to explore nanocarriers as a means to deliver medicine against parasitic infections.

## 1. Introduction

The interest in nanotechnology is on the rise, especially concerning pharmaceutical nanocarriers, which include nanocapsules, nanospheres, nanoemulsion, and nano-sized vesicles [1]. The ability of nanocarriers to overcome the limitations of conventional drug regimens may allow for the development of more efficient treatments [1,2]. In particular, this emerging field has made limited advances against rare but serious and sometimes fatal parasitic infections [3,4,5,6,7,8,9,10]. Given the ubiquitous nature of amoebae, it is anticipated that the number of infections due to free-living pathogenic amoebae will rise in the coming years [11,12,13]. These amoebae enter the brain and often lead to death; hence any effective drugs need to traverse the microvessels to enter the brain to eradicate the residing parasites, coupled with minimal toxicity [14]. The current therapeutic regimen for *B. mandrillaris* infections comprises a combinational approach of various antimicrobials, such as fluconazole, flucytosine, sulfadiazine, pentamidine, clarithromycin or azithromycin, and miltefosine, and for *N. fowleri*, the therapy often comprises of amphotericin B, fluconazole, rifampin, miltefosine, azithromycin, and dexamethasone [11,15,16]. These antimicrobials depict often detrimental side effects, such as nephrotoxicity, as the concentration of drugs required is very high to eradicate the amoebae effectively in the CNS [12,17,18,19]. 

Previous studies have utilized nanotechnology versus pathogenic free-living amoebae, including gold and silver nanoparticles as well as green synthesized nanoparticles stabilized by gums (derived from plants), which showed promising activities [8,20,21]. Furthermore, novel carriers/nanoparticles may facilitate the passage of nanoconjugates across the blood brain barrier. Moreover, the incorporation of nanotechnology and novel nanocarriers in neuroimaging techniques, such as magnetic resonance imaging (MRI), has potential for diagnosing. A recent example is the use of superparamagnetic iron oxide nanoparticles, which were shown to act as contrasting agents, thereby increasing sensitivity of the images in the MRI [22]. Another interesting study revealed that nanosuspensions coated with polysorbate and sodium cholate increased the delivery of amphotericin B and inhibited *B. mandrillaris* in vitro, though were less potent in vivo [23]. To this end, various compounds, such as curcumin and amphotericin B, were conjugated with novel nanocarriers and tested against the brain-eating amoebae, *N. fowleri* and *B. mandrillaris,* for their amoebicidal, cytotoxic and parasite-mediated host cell death properties. The novel nanocarriers utilized in this study comprise of lactobionic acid-coated magnetic nanoparticles loaded with curcumin and amphotericin B along with lecithin-amphotericin B based liposomes, and we speculate that these nanocarriers will be more effective in facilitating drug delivery with limited host cell toxicity.

## 2. Materials and Methods

Lactobionic acid dicyclohexyl carbodimide (DCC), 4-dimethyl aminopyridine (DMAP), ammonium hydroxide (NH_4_OH), cinnamic acid (CA), 3-aminopropyl silane (APT), ferrous sulfate heptahydrate (FeSO_4_·7H_2_O), ferric sulfate hexahydrate (Fe_2_(SO_4_)_3_·6H_2_O), were purchased from Sigma Aldrich (Steinheim am Albuch, Germany). Lecithin was purchased from Lipoids, while curcumin was procured from Carl Roth (Karlsruhe, Germany), and amphotericin B was purchased from Sigma Aldrich (Germany). All solvents used were HPLC grade and obtained from Fischer scientific, through a local supplier.

### 2.1. Nanoparticle Preparation

A solvent diffusion technique was adopted for the preparation of amphotericin B loaded with lecithin (LEC−AMPB) nanoparticles, which has been described in detail previously [24,25]. Preparation of lactobionic acid (LBA), curcumin (CUR) and amphotericin B (AMPB) magnetic nanoparticles (MNP), namely: MNP−LBA, MNP−LBA−CUR and MNP−LBA−AMPB, was accomplished via functionalization of LBA on the surface of the nanoparticles, as detailed previously [24]. Subsequently, an analysis via ultraviolet–visible (UV-VIS) spectrophotometer at 405 nm for AMPB and 424 nm for CUR was conducted. The chemical interactions of the synthesized nanoparticles were determined via an FTIR spectrophotometer (Shimadzu, Koyoto, Japan).

### 2.2. Morphology, Hydrodynamic Diameter, and Polydispersity Index (PDI)

A zetasizer was utilized to analyze the average size and PDI, as previously described [24]. Briefly, the nanoparticles were diluted with distilled water and were transferred to a cuvette, and placed in a sample holder to perform the studies at room temperature. The surface morphology of the nanoparticles was carried out using AFM microscopy. In general, 10 µL of nanosuspension was dropped on a mica slide and allowed to dry in a dust-free environment and then mounted onto a microscope for imaging at non-contact mode.

### 2.3. Efficient Drug Entrapment Determination

The drug loading efficiency of the nanoparticles was accomplished as previously described. Briefly, nanosuspensions were subjected to centrifugation at 12,000× *g* rpm for 15 min to separate the nanoparticles. Next, the nanoparticles were diluted successively and analyzed at their respective wavelengths using a UV-VIS spectrophotometer [24]. Entrapment efficiency was determined using the equation below [26]:

$$\%\;\mathrm{Drug}\;\mathrm{Entrapment}=\frac{\mathrm{Amount}\;\mathrm{of}\;\mathrm{drug}\;\mathrm{used}\;-\;\mathrm{unloaded}\;\mathrm{drug}}{\mathrm{Amount}\;\mathrm{of}\;\mathrm{drug}\;\mathrm{used}}\times 100$$

### 2.4. HeLa Cells

Cervical cancer cells derived from Henrietta Lacks, known as HeLa cells, were procured from the American Type Culture Collection (ATCC CCL-2) and cultivated in T-75 tissue culture flasks containing 10 mL of RPMI-1640, 1% Penicillin-Streptomycin (Pen-Strep), 1% minimum essential medium amino acids and 1% L-glutamine in a humidified environment (more than 95% humidity) of 5% CO_2_ at 37 °C. After the media was removed aseptically, the cells were detached using 2 mL of trypsin EDTA. The cells in the culture were then centrifuged for 5 min at 2500× *g* [27,28]. The cell pellet was resuspended in the medium, and then transferred to 24-well or 96-well plates and employed in a variety of assays.

### 2.5. Naegleria fowleri Culture

*Naegleria fowleri* (strain HB1; ATCC 30174) was procured from ATCC [6]. The parasites (5 × 10^5^ amoebae) were added to confluent monolayers of HeLa cells, grown in T-75 flasks, which served as a food source for the *N. fowleri*_._ After 48 h, the parasites had consumed the HeLa cells and increased to approximately 1–5 × 10^6^ amoebae of which 95% were in the trophozoite form. These trophozoites were then used for subsequent assays as described previously [8,19]. 

### 2.6. Balamuthia mandrillaris Culture

*B. mandrillaris* (strain ATCC 30174) was attained from ATCC. *B. mandrillaris* (5 × 10^5^ amoebae) were added to confluent monolayers of HeLa cells, grown in T-75 flasks, which served as a food source for the *B. mandrillaris*. After 48 h, the *B. mandrillaris* had consumed the HeLa cells and increased to approximately 1–5 × 10^6^ amoebae of which 95% were in the trophozoite form. These trophozoites were then used for subsequent assays as before [8,19].

### 2.7. Amoebicidal Assay

Experimentation via assays to comprehend the cidal properties of the compounds and their nanoformulations were carried out as explained previously [8,19]. In short, 2 × 10^5^ parasites were incubated with drugs and nanoconjugated-drugs and the final volume was adjusted to 200 µL for 24 h at 37 °C, in a 5% CO_2_ with 95% humidity. Parasites incubated without drugs were used as negative controls and parasites incubated with 0.25% SDS were used as positive controls. Following 24 h incubation, Methylene blue (0.1%) was added to each well and hemocytometer counting was carried out to determine the viable amoebae. The basis of this assay is that the dye penetrates the cell membrane of the dead parasites and they appear stained (i.e., dark blue), while live parasites exclude the dye and the amoebae appear unstained.

### 2.8. Cytotoxicity Assays

To determine whether the drugs or drugs-conjugated with nanoparticles produce human cell damage, cytotoxicity assays were performed. Briefly, HeLa cells were grown in 96-well plates, and the cells were treated with the various compounds/formulations for different intervals of time (up to 24 h) in a 95% humidifying incubator, with 5% CO_2_, and kept at 37 °C [19,24]. A cell cytotoxicity kit was utilized for lactate dehydrogenase (LDH) enzyme determination [29]. For the positive control, 1% Triton X-100 was added to each well and the cells were incubated for 45 min at 37 °C. For the negative control, HeLa cells were incubated with RMPI-1640 alone. In a 96-well plate, an equivalent volume of LDH substrate was added with a similar volume of experimental cell supernatant. The plate was incubated in the dark at room temperature and then subjected to a multi-plate reader. At 490 nm, the absorbance was determined, and the percent cytotoxic effect was calculated using the following formula: % Cell cytotoxicity = ((Sample value − negative control)/(Positive control − negative control)) × 100

### 2.9. Cytopathogenicity Experiments 

Cytopathogenicity assays were accomplished to comprehend the parasite-mediated host cell death and the inhibitory effects of the drugs [8,19]. In short, 2 × 10^5^ parasites were incubated with different drugs and their nanoformulations and placed for 2 h at 37 °C in a 5% CO_2_ incubator with 95% humidity. Next, drug-treated parasites were then placed in 96-well plates containing HeLa cell monolayers. Next, the 96-well plates were incubated for various intervals of time (up to 24 h) in a 95% humidifying incubator, with 5% CO_2_, and kept at 37 °C. Following this incubation, the supernatants were collected and host cell cytotoxicity was determined as described above. The positive and negative controls included HeLa cells with Triton x-100 (1%), whereas the negative control included HeLa cells incubated with RPMI-1640 alone.

### 2.10. Statistical Analyses

All statistical comparisons were conducted using a two-sample *t*-test, with two-tailed distribution. The data are presented as the mean ± standard error of several experiments. *p* ≤ 0.05 was considered as the statistical significance level. 

## 3. Results

### 3.1. Effective Preparation of AMPB−Lecithin Nanoformulations

**Scheme 1:**
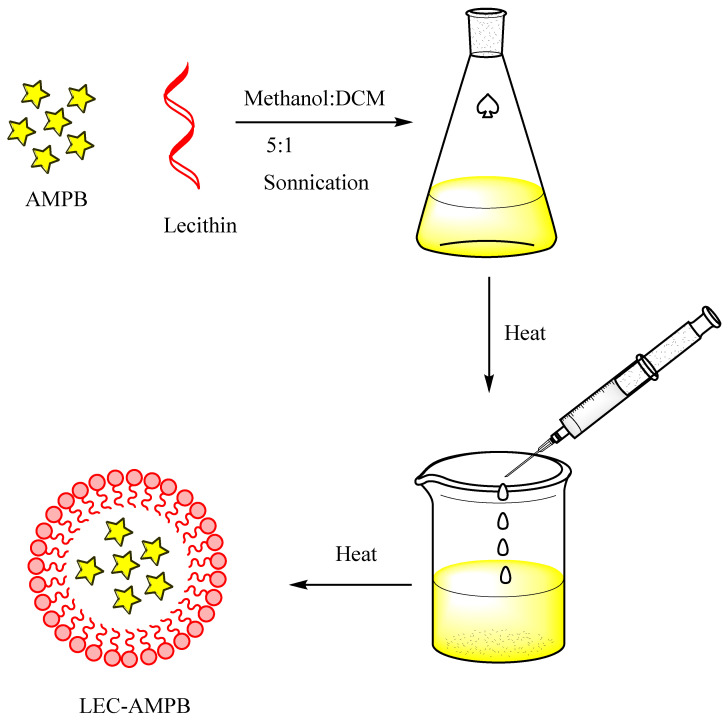
General scheme for the preparation of LEC−AMPB NPs.

**Figure 1:**
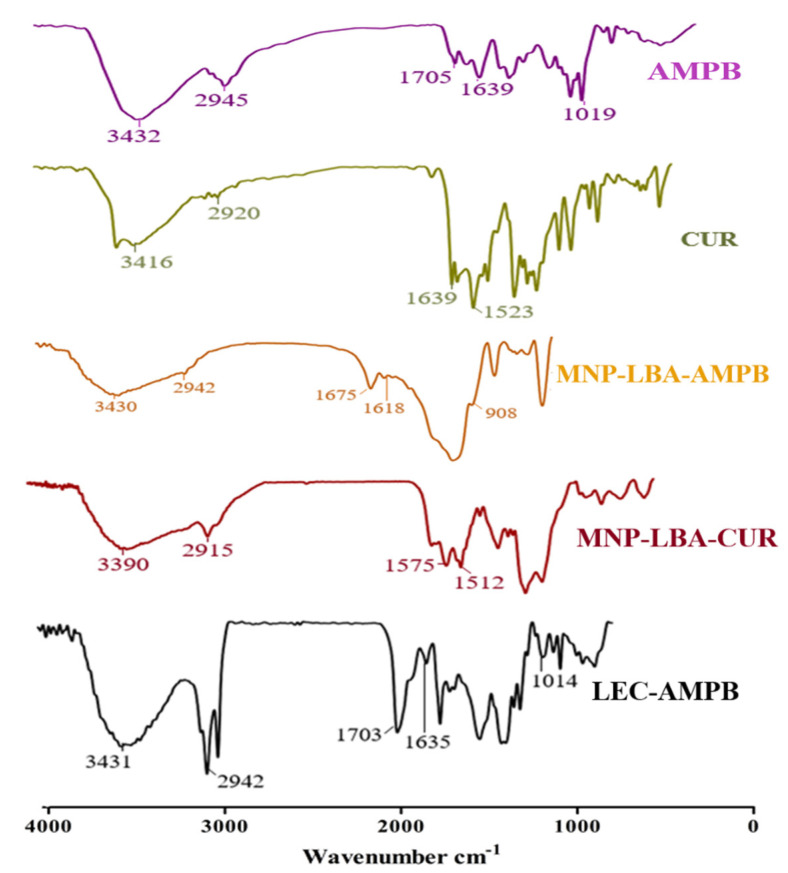
FTIR spectra of AMPB, CUR, MNP−LBA−AMPB, MNP−LBA−CUR and LEC−AMPB.

As detailed earlier, the AMPB was entrapped in lecithin nanoparticles (Scheme 1), and then freeze-dried at −20 °C, and reconstituted in SDS to improve the colloidal stability of the LEC−AMPB NPs. The stacking of AMPB was characterized by FTIR spectroscopy. The FTIR spectra of AMPB showed an absorption of ~1705 cm^−1^ and 1643 cm^−1^, consistent with (C=O) and (C=C) moiety [30]. Stretching frequency at 3433 cm^−1^, which is consistent with OH stretching, was observed (Figure 1). The FTIR spectra of LEC−AMPB revealed nearly indistinguishable variation in the drug frequencies, which portrayed that the AMPB was chemically stable within the LEC−AMPB NPs [30].

### 3.2. Effective Formulation of MNP−LBA, MNP−LBA−CUR and MNP−LBA−AMPB

**Scheme 2:**
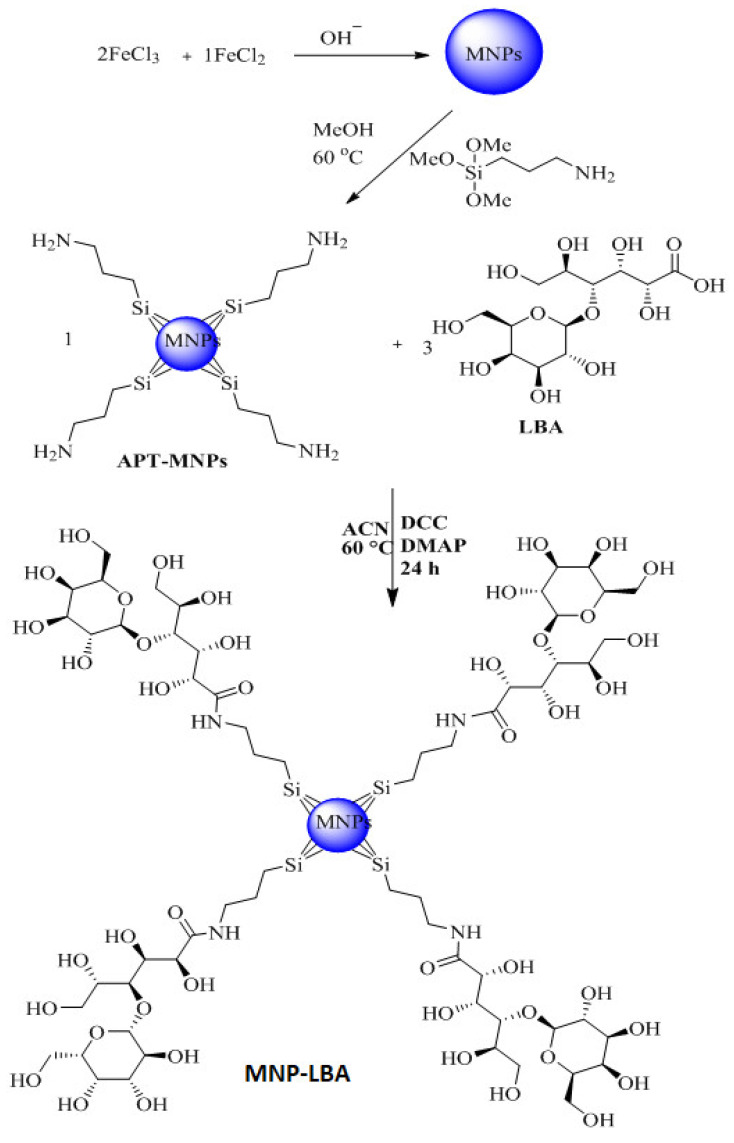
Synthetic scheme for the preparation of MNP−LBA.

**Figure 2:**
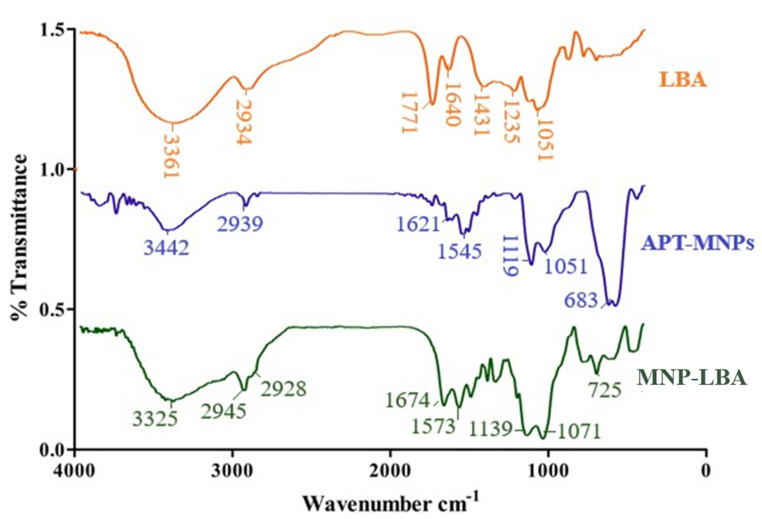
FTIR spectra of LBA, MNP−LBA and APT−MNPs.

The amino-functionalized magnetic nanoparticles and LBA conjugated analogue are depicted in Scheme 1 and Scheme 2. NH_2_ bending and stretching vibrations at 3442 cm^−1^ and 1621 cm^−1^ were seen [31]. The stretching frequency of 2939 cm^−1^ is known to correspond to the propyl group (Figure 2). However, following functionalization with LBA, the frequency was 1674 cm^−1^ and 1071 cm^−1^, corresponding to amide C=O and C-O-C, (Figure 2), thus validating the effective formulation of LBA on magnetic nanoparticles. The increased absorption at 3325 cm^−1^ indicates the hydroxyl group of functionalized ligands [32] and the successful formation of MNP−LBA. The FTIR spectrum of CUR depicted a peak at 3416 cm^−1^ for the OH stretching. C-H, C=C and C=O absorptions were depicted at 2920 cm^−1^, 1639 cm^−1^ and 1523 cm^−1^, respectively [33]. In the MNP-LBA-CUR formulation, a variation was detected for the OH stretching as a peak shifted to 3390 cm^−1^, while the C=C and C=O stretching frequency were shifted to 1575 cm^−1^ and 1512 cm^−1^, respectively (Figure 1), revealing entrapment of CUR within the MNP−LBA (Scheme 2).

AMPB revealed absorption at 1705 cm^−1^ and 1643 cm^−1^ corresponding to the (C=O) and (C=C) moiety [30]. A stretching frequency at 3433 cm^−1^ was observed (Figure 1). The MNP−LBA−AMPB revealed variation in the absorption frequencies, a peak at 1705 cm^−1^ of carboxylic acid (C=O), which was shifted to 1675 cm^−1^ and a peak at 1643 cm^−1^ was shifted to 1618 cm^−1^. The peak at 1019 cm^−1^ of the acetal bond was shifted to 908 cm^−1^ (Figure 1). The absorption at 3433 cm^−1^ of OH was shifted to 3430 cm^−1^ (Figure 1). 

### 3.3. Nanoformulations Properties 

**Figure 3:**
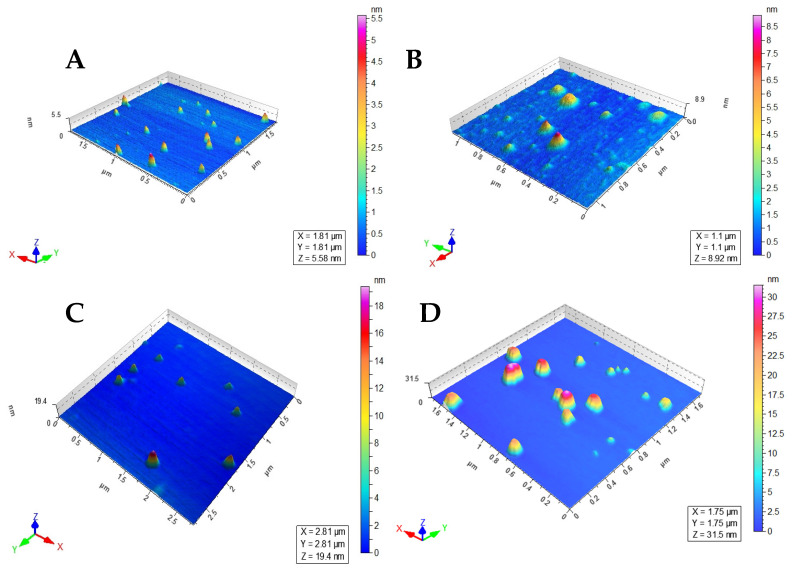
Atomic force microscopic images of (**A**) MNP−LBAs, (**B**) MNP−LBA−AMPB, (**C**) MNP−LBA−CUR and (**D)** LEC−AMPB.

The size of the LEC−AMPB, MNP−LBA, MNP−LBA−CUR and MNP−LBA−AMPB are described in Table 1. The increase in size of the MNP−LBA−CUR and MNP−LBA−AMPB is likely due to the drug incorporated into the cavity of MNP-LBA [26]. In the case of LEC−AMPB, the size was found to be 144 ± 16.2, suggesting its potential for biomedical applications [30]. The PDI revealed a dispersion of the nanoformulation, and the 0.5 value of PDI is indicative of size broadening [34]. The PDI values of LEC−AMPB, MNP−LBA, MNP−LBA−CUR and MNP−LBA−AMPB are shown in Table 1. Drug-containing formulations possess uniform colloidal dispersibility, suggestive of increased colloidal stability nanoformulations, as confirmed with an analysis of the literature [35]. The nanoparticles revealed near spherical morphology, indicative of nanoformulations stability, and confirmed via AFM (Figure 3).

### 3.4. Drug Entrapment Efficiency

Higher drug loading is needed for effective drug delivery. In addition, a higher amount of drug loading increases the sustainability of the nanoformulation for a prolonged extent [30]. The drug entrapment efficiency of LEC−AMPB was found to be 86.24 ± 1.32%. Increased drug loading may be due to the lipophilic nature of both AMPB and the lecithin molecules [30]. For MNP−LBA−CUR and MNP−LBA−AMPB, the loading efficiency was 41 ± 3.2% and 79.4 ± 0.69%, respectively. Higher amounts of drug loading are likely possible because of hydrophobic cavities and increased secondary interactions within the LBA moieties [36], which may favor the encapsulation of hydrophobic drugs [37]. 

### 3.5. Nanocarrier-Conjugated Curcumin and Amphotericin B Showed Significant Amoebicidal Effects against Balamuthia mandrillaris and Naegleria fowleri

**Figure 4:**
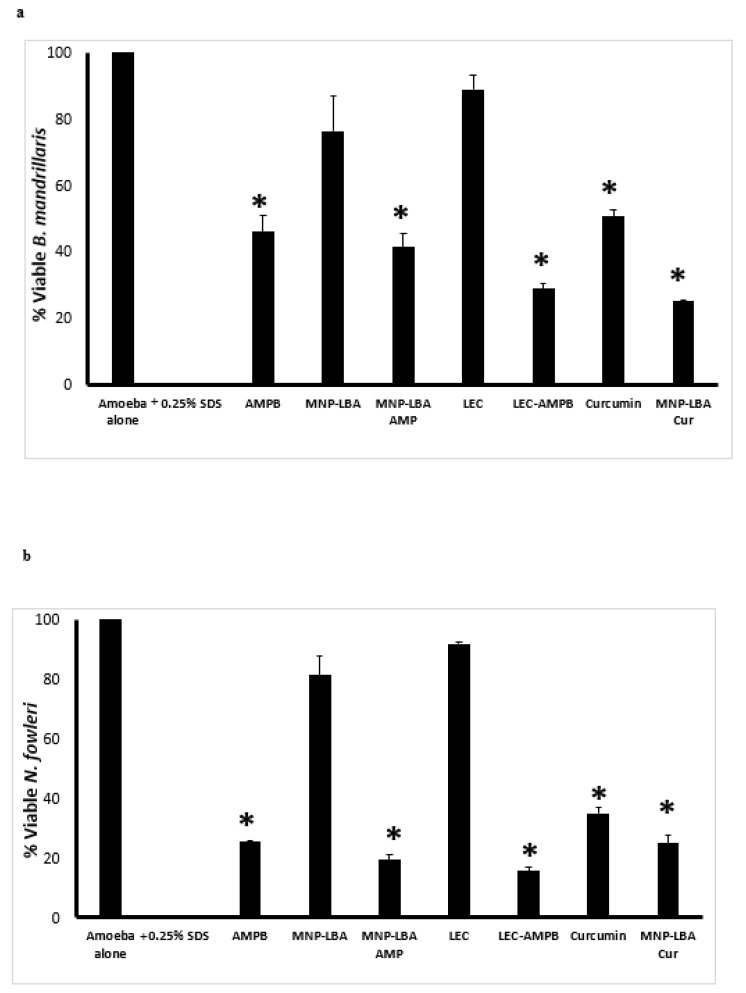
Nanocarrier drug conjugates showed significant amoebicidal effects against *Balamuthia mandrillaris* and *Naegleria fowleri*. Significant antiamoebic effects of the majority of compounds against *B. mandrillaris* (**a**) and *N. fowleri* (**b**) were observed after a 24 h incubation period. All data is illustrative as the mean ± standard error. Additionally, *p*-values were determined through the conduction of a two sample *t*-test, (*) is ≤ 0.05. AMPB is Amphotericin B; LEC is Lecithin; LBA is lactobionic acid; CUR is curcumin; MNP is magnetic nanoparticles.

To comprehend the antiparasitic properties of drugs alone and nanocarrier drug conjugates, amoebicidal assays were conducted against *B. mandrillaris* and *N. fowleri*. The results revealed that AMPB alone exhibited up to 54% cidal effects against *B. mandrillaris* at 100 µg/mL and these effects were enhanced up to 60% and 70% when conjugated with MNP−LBA and LEC, respectively (Figure 4a) (*p* < 0.05). Of interest, nanocarriers alone (MNP−LBA and LEC) showed no significant effects against *B. mandrillaris*. Similarly, curcumin alone exhibited up to 50% cidal effects against *B. mandrillaris* at 100 µg/mL and these effects were enhanced up to 75% when conjugated with MNP−LBA (Figure 4a) (*p* < 0.05). 

When tested against *N. fowleri*, AMPB alone exhibited up to 75% cidal effects at 100 µg/mL and these effects were enhanced up to 80% and 85% when conjugated with MNP−LBA and LEC, respectively (Figure 4b) (*p* < 0.05). Of interest, nanocarriers alone (MNP−LBA and LEC) showed no significant effects against *N. fowleri*. Similarly, curcumin alone exhibited up to 65% cidal effects against *N. fowleri* at 100 µg/mL and these effects were enhanced up to 75% when conjugated with MNP−LBA (Figure 4b) (*p* < 0.05). 

### 3.6. Nanocarrier-Conjugation Reduced Curcumin and Amphotericin B cytotoxicity to Human Cells

**Figure 5:**
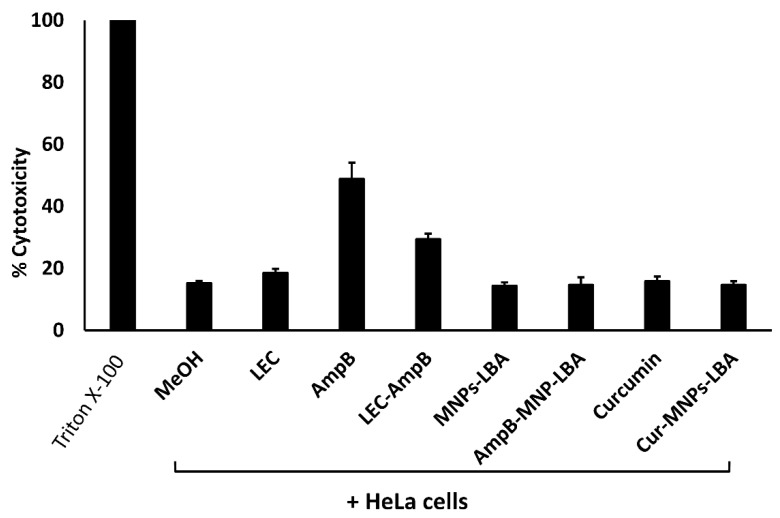
Conjugation with nanocarriers reduced cytotoxicity against human cell lines. Confluent monolayers of HeLa cells were treated with 100 µg/mL of various compounds, as described in Materials and Methods. AMPB is Amphotericin B; LEC is Lecithin; LBA is lactobionic acid; CUR is curcumin; MNP is magnetic nanoparticles.

To evaluate the cytotoxicity of drugs versus human cells, LDH cytotoxicity experiments were accomplished using HeLa cells. Referring to the ISO-10993-5, if the cell survival following treatment is >80%, then the compounds are safe, 60–80% suggests limited cytotoxic activity is present, between 40% to 60% suggests moderately toxic activity is present, and if cell viability is lower than 40% then the compounds are considered toxic [38]. The cytotoxic activities of the solvent alone (methanol), and the nanocarriers alone (LEC, MNP−LBA) were less than 20%. In contrast, AMPB alone showed cytotoxic effects of up to 49%. When conjugated with LEC and MNP−LBA, the cytotoxic effects of AMPB were reduced to 29% and 15%, respectively (Figure 5). However, curcumin alone or curcumin conjugated with MNP−LBA showed cytotoxic effects of 15.8% and 14.6%, respectively (Figure 5). 

### 3.7. Nanocarrier-Conjugated Curcumin and Amphotericin B Reduced Human Cell Cytopathogenicity Caused by Balamuthia mandrillaris and Naegleria fowleri

**Figure 6:**
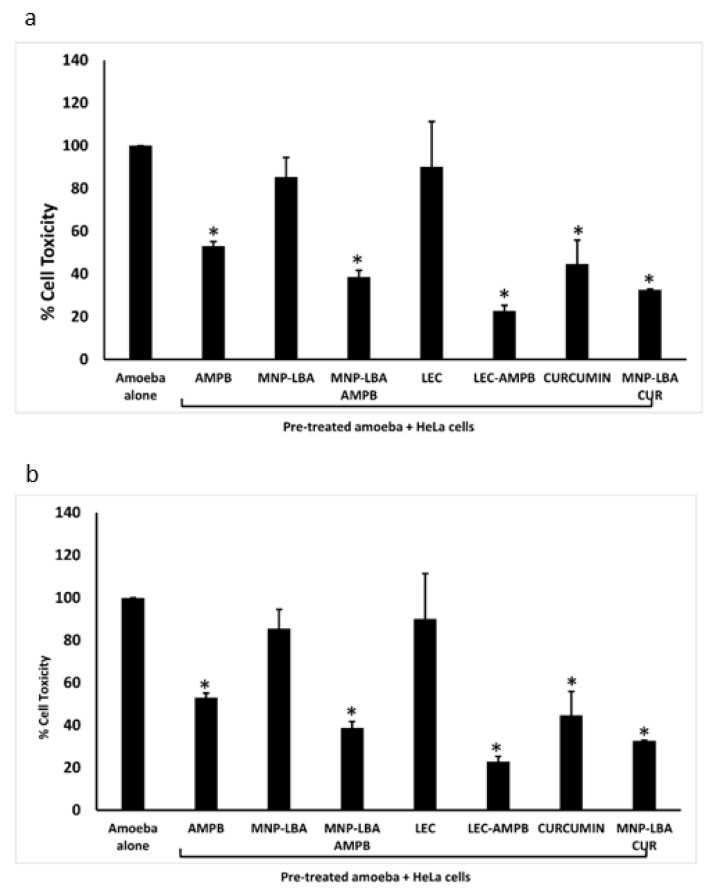
Nanocarrier drug conjugates reduced amoeba-mediated host cell cytopathogenicity (**a**) The compounds reduced *Balamuthia mandrillaris* mediated cytotoxicity against human cells and (**b**) the compounds reduced *Naegleria fowleri* mediated cytotoxicity against human cells. All data is illustrative as the mean ± standard error. Additionally, *p*-values were determined through the conduction of a two sample *t*-test, (*) is ≤ 0.05. AMPB is Amphotericin B; LEC is Lecithin; LBA is lactobionic acid; CUR is curcumin; MNP is magnetic nanoparticles.

Cytopathogenicity experiments were carried out to comprehend parasite-mediated host cell damage. The experiments portrayed that following pre-treatment with the drug nanocarriers, amoebae-mediated cell damage was diminished significantly (Figure 6) (*p* < 0.05). When treated with AMPB alone, *B. mandrillaris*-mediated cell damage was diminished up to 50% (Figure 6a). However, nanocarrier drug conjugates reduced cell damage to 38% and 31% using MNP−LBA−AMPB and LEC−AMPB, respectively. Similarly, when treated with curcumin alone, *B. mandrillaris*-mediated cell damage was 45% (Figure 6a), but nanocarrier drug conjugates reduced cell damage to 32% using MNP−LBA−CUR (Figure 6a).

For *N. fowleri*, the results revealed that when treated with AMPB alone, cell damage was reduced up to 37% (Figure 6b). However, nanocarrier drug conjugates reduced cell damage to 27% and 22% using MNP−LBA−AMPB and LEC−AMPB, respectively. Similarly, when treated with curcumin alone, cell damage was reduced up to 38% (Figure 3b), but nanocarrier drug conjugates reduced cell damage to 28% using MNP−LBA−CUR (Figure 6b).

## 4. Discussion

Pathogenic amoebae, *Naegleria fowleri* and *Balamuthia mandrillaris* are unicellular protists distributed across the environment. Given opportunity and access, they can produce infections of the CNS that almost always result in death [39,40]. It is distressing that the mortality rate has remained more than 90% despite advances in antimicrobial chemotherapy and supportive care [11,41]. In part, this is due to an incomplete understanding of the pathogenesis and pathophysiology of the disease as well as a lack of availability of targeted and effective chemotherapeutic agents that can be delivered to the target site without harming the host cells. Hence, current treatment options are a hit and miss approach that consists of often repurposed drugs, including a combinatorial approach comprising of antitumor agents, antiparasitic drugs, antivirals, antibacterials, etc., and despite this, the prognosis remains extremely poor [15,16]. Additionally, amphotericin B is an antifungal polyene antibiotic and often used against primary amoebic meningoencephalitis (PAM) due to *N. fowleri*; however, it is found to possess serious side effects, such as liver or nephrotoxicity [19]. This is due to the fact that an adequate concentration of the drug has to be given using an intravenous route to ensure the minimum inhibitory concentration is achieved at the target site, i.e., the CNS, to kill the residing parasite. In the absence of effective drug delivery, there is a pressing urgency to design novel formulations that are capable of passing the blood brain barrier at physiologically safe concentrations to treat the brain infection, while remaining non-toxic for the patient [42]. In this regard, nanotechnology may be a possible strategy to overcome the limitations of conventional drug regimens [1]. This is not surprising, as previous literature suggests that the efficiency of polymeric nanocarriers is augmented and these were found to act as functional carriers of compounds to the CNS [14]. Thus, in this study, the possibility of enhancing the pharmacokinetics and therapeutic efficacy of compounds versus amoebae was determined.

To this end, amphotericin B and curcumin were conjugated with novel nanocarriers and their antiamoebic effects were evaluated. Of note, most of the compounds, when conjugated with novel nanocarriers, depicted antiamoebic effects, such as MNP−LBA, MNP−LBA−AMPB, LEC, curcumin and MNP−LBA−CUR; however, it is MNP−LBA−CUR that was found to be of more value. The curcumin-loaded nanoparticle exhibited amoebicidal properties greater than that of curcumin alone. Furthermore, the CUR-MNP-LBA compound possessed the least MIC_50_ value for both *B. mandrillaris* and *N. fowleri* at 62.60 µg/mL and 61.57 µg/mL, respectively. Moreover, MNP−LBA−CUR demonstrated the highest protection against amoebae-mediated host cell cytotoxicity, i.e., a reduction in viable *B. mandrillaris* to 36.6% and a reduction in viable *N. fowleri* to 37.2%. Additionally, five of the compounds exhibited negligible toxic effects against human cells tested in this study, whereas two compounds, LEC and LEC−AMPB, exhibited weak toxicity, and one compound, AMPB, exhibited moderate toxicity, indicative of their potential use versus amoebic infections.

Curcumin is a phenolic compound obtained from turmeric. It is used as a coloring agent, spice, and food preservative [43]. This compound is found to possess antimicrobial, antioxidant, antitumor, and anti-inflammatory properties. However, curcumin has low solubility and poor bioavailability, and as a result, the development of nanoparticles to carry the compound can enhance its effects and overcome delivery challenges. Previously, curcumin effects versus *Acanthamoeba castellanii* were determined and the results showed that curcumin-loaded nanocarriers could serve as a potential therapeutic source against *A. castellanii* [44]. Furthermore, in another previous study, dimethoxycurcumin exhibited promising activity against *A. castellanii.* The possible mechanism for the amoebicidal activity of dimethoxycurcumin were suggested to be due to the release of proteases from the intracellular stores of *A. castellanii* [28], which may explain the findings observed in the present study. 

A previous study examined the effects of the conjugation of amphotericin B on *N. fowleri,* revealing encouraging results; however, in this study, silver nanoparticles were utilized for the conjugation, and the cytotoxicity of the compounds alone was not evaluated [8]. Furthermore, until now, conjugated curcumin and amphotericin B have not been evaluated on *B. mandrillaris*, for which the number of reported cases is evident [45,46,47]. The nanocarriers utilized in this study also revealed greater drug loading efficiency, thus showing their capability for higher drug release to the desired target site [30]. Nonetheless, forthcoming research is warranted to understand the mechanism of action and to determine the appropriate doses, for safety and efficacy, for the clinical use of the aforementioned drugs in the treatment of infections due to *B. mandrillaris* and *N. fowleri*. Further studies also need to be conducted against the cyst stage of pathogenic amoebae. Additionally, a three-dimensional model of the blood brain barrier should be utilized to test these particular nanocarrier drug formulations for their efficiency in passing through the barrier. Finally, in vivo studies will be needed in order to bring these novel nanocarrier conjugated compounds to the clinic for use against these devastating and fatal infections.

## Figures and Tables

**Table 1:** Average size and PDI of drug loaded LEC−AMPB, MNP−LBA, MNP−LBA−CUR and MNP−LBA−AMPB nanocomposites.

Nanoparticles	Average Size (nm)	PDI
LEC−AmpB	144 ± 16.2	0.26 ± 0.050
MNP−LBA	171.9 ± 2.23	0.24 ± 0.015
MN−-LBA−CUR	230.1 ± 3.13	0.32 ± 0.040
MNP−LBA−AMPB	189.7 ± 7.89	0.35 ± 0.020

## Data Availability

Data is contained within the article.
